# Familial hypertriglyceridemia: an entity with distinguishable features from other causes of hypertriglyceridemia

**DOI:** 10.1186/s12944-021-01436-6

**Published:** 2021-02-15

**Authors:** Ivette Cruz-Bautista, Alicia Huerta-Chagoya, Hortensia Moreno-Macías, Rosario Rodríguez-Guillén, María Luisa Ordóñez-Sánchez, Yayoi Segura-Kato, Roopa Mehta, Paloma Almeda-Valdés, Lizeth Gómez-Munguía, Ximena Ruiz-De Chávez, Ximena Rosas-Flota, Arali Andrade-Amado, Bárbara Bernal-Barroeta, María Guadalupe López-Carrasco, Luz Elizabeth Guillén-Pineda, Angelina López-Estrada, Daniel Elías-López, Alexandro J. Martagón-Rosado, Donají Gómez-Velasco, Cesar Ernesto Lam-Chung, Omar Yaxmehen Bello-Chavolla, Fabiola Del Razo-Olvera, Lucely D. Cetina-Pérez, José Luis Acosta-Rodríguez, María Teresa Tusié-Luna, Carlos A. Aguilar-Salinas

**Affiliations:** 1 Unidad de Investigación de Enfermedades Metabólicas, Instituto Nacional de Ciencias Médicas y Nutrición Salvador Zubirán, Vasco de Quiroga 15, Tlalpan, 14080 Mexico City, Mexico; 2 Departamento de Endocrinología y Metabolismo, Instituto Nacional de Ciencias Médicas y Nutrición Salvador Zubirán, Vasco de Quiroga 15, Tlalpan, 14080 Mexico City, Mexico; 3 Unidad de Biología Molecular y Medicina Genómica, Instituto Nacional de Ciencias Médicas y Nutrición Salvador Zubirán e Instituto de Investigaciones Biomédicas de la UNAM, Mexico City, Mexico; 4 CONACyT. Instituto Nacional de Ciencias Médicas y Nutrición Salvador Zubirán, Mexico City, Mexico; 5 Departamento de Economía, Universidad Autónoma Metropolitana, Mexico City, Mexico; 6 Tecnológico de Monterrey, Escuela de Medicina y Ciencias de la Salud, Ave. Morones Prieto 3000, 64710 Monterrey, NL Mexico; 7 Research Division, Instituto Nacional de Geriatría, Mexico City, Mexico; 8 Departamento de Oncología Médica, Instituto Nacional de Cancerología, Mexico City, Mexico; 9 Departamento de Biotecnología Agrícola, Instituto Politécnico Nacional, Mexico City, Mexico

**Keywords:** Familial hypertriglyceridemia, Mexicans, Primary dyslipidemias, Chylomicronemia, FGF-21, ANGPTL3, Apolipoprotein B

## Abstract

**Background:**

Familial hypertriglyceridemia (FHTG) is a partially characterized primary dyslipidemia which is frequently confused with other forms hypertriglyceridemia. The aim of this work is to search for specific features that can help physicians recognize this disease.

**Methods:**

This study included 84 FHTG cases, 728 subjects with common mild-to-moderate hypertriglyceridemia (CHTG) and 609 normotriglyceridemic controls. All subjects underwent genetic, clinical and biochemical assessments. A set of 53 single nucleotide polymorphisms (SNPs) previously associated with triglycerides levels, as well as 37 rare variants within the five main genes associated with hypertriglyceridemia (i.e. LPL, *APOC2, APOA5, LMF1* and *GPIHBP1)* were analyzed. A panel of endocrine regulatory proteins associated with triglycerides homeostasis were compared between the FHTG and CHTG groups.

**Results:**

Apolipoprotein B, fibroblast growth factor 21(FGF-21), angiopoietin-like proteins 3 (ANGPTL3) and apolipoprotein A-II concentrations, were independent components of a model to detect FHTG compared with CHTG (AUC 0.948, 95%CI 0.901–0.970, 98.5% sensitivity, 92.2% specificity, *P* < 0.001). The polygenic set of SNPs, accounted for 1.78% of the variance in triglyceride levels in FHTG and 6.73% in CHTG.

**Conclusions:**

The clinical and genetic differences observed between FHTG and CHTG supports the notion that FHTG is a unique entity, distinguishable from other causes of hypertriglyceridemia by the higher concentrations of insulin, FGF-21, ANGPTL3, apo A-II and lower levels of apo B. We propose the inclusion of these parameters as useful markers for differentiating FHTG from other causes of hypertriglyceridemia.

**Supplementary Information:**

The online version contains supplementary material available at 10.1186/s12944-021-01436-6.

## Introduction

Hypertriglyceridemia (HTG) is a common medical condition associated with abnormal very-low-density lipoprotein (VLDL) and chylomicron particle number and composition and in certain cases, is associated with increased cardiovascular risk [1]. According to the European Society of Cardiology and European Atherosclerosis Society, mild-to- moderate HTG is defined by triglycerides levels > 150-880 mg/dL, and severe HTG refers to levels > 880 mg/dL [2, 3]. The prevalence of HTG varies among ethnic groups [4, 5]; it is highly prevalent in Hispanics. In Mexico, the prevalence of mild-to-moderate hypertriglyceridemia is around 31%, with ~ 5% of the Mexican population showing severe HTG [6].

Familial hypertriglyceridemia (FHTG) is a form of HTG, in which the clinical and genetic spectrum has not been elucidated [7]. FHTG is frequently confused with other causes of HTG. This includes familial chylomicronemia syndrome (FCS) [8, 9], a condition caused by mutations in the lipoprotein lipase (*LPL*) gene or other genes that regulate the function of this enzyme (*i.e APOC2* and the *LMF1* (lipase maturation factor 1)) [10–14]. This also includes conditions characterized by common mild-to-moderate hypertriglyceridemia (CHTG), such as familial combined hyperlipidemia (FCHL). The diagnosis of this dyslipidemia is suggested by a fluctuating lipid profile, apolipoprotein B (apo B) levels >90th population percentile for age and gender and a first-degree family history of premature cardiovascular disease [15–17].

FHTG was described by Glueck and co-workers in 1973. It is the most common form of primary isolated hypertriglyceridemia. Its prevalence in Caucasian populations has been estimated to be around 0.5–1% [18]. In Mexico, the exact prevalence of FHTG is unknown, although it is frequently diagnosed associated with pancreatitis. The inheritance of this disease is autosomal dominant with variable penetrance, however, the genes involved are still largely unknown. A genome-wide association study (GWAS) in the Mexican population, identified genes associated with triglyceride levels in patients with moderate hypertriglyceridemia [19]. As yet, there is no information about the contribution of these genes in FHTG [20]. The principle pathophysiological defect appears to be a deficit in the catabolism of very-low density lipoproteins (VLDLs). The number of particles is relatively stable, but the triglyceride content is increased. Thus, there is an increase in VLDL particle size with no concomitant elevation of apo B concentration [21]. Large VLDL particles are not an optimum substrate for LPL, hence, hydrolysis is slow. A simultaneous decrease in high-density lipoprotein cholesterol (HDL-C) is frequently found. FHTG is characterized by the presence of fasting plasma triglyceride (TG) levels >95th population percentile for age and gender, as well as normal to low total cholesterol levels [18]. Therefore, the biochemical presentation of FHTG is characterized by variable levels of triglycerides (200–700 mg/dL) and the presence of triglyceride rich VLDL particles (hyperlipidemia (HLP) type 4). When FHTG coexists with other comorbidities, such as type 2 diabetes (T2D), the metabolic syndrome (MS), or environmental factors such as increased alcohol intake or a high fat diet [22–26], the levels of triglycerides can reach values of over 1000 mg/dL with a TG/cholesterol ratio of 5:1, accompanied by the presence of chylomicrons (HLP type 5). Hence, this condition is associated with a life-threatening risk of acute pancreatitis [27]. The normal apo B levels encountered in FHTG, even when TG levels are below 500 mg/dL allows it to be distinguised from FCHL [17].. Although, some authors have suggested that FHTG is not different from other oligogenic/polygenic forms of hypertriglyceridemia (e.g. FCHL) [28]. Diagnosis of FHTG is confirmed when the proband and one or more of their family members show a similar lipid pattern [20, 22]. The atherogenicity of FHTG is a subject of great controversy; Mendelian randomization studies have demonstrated a causal role of the triglyceride and triglyceride-rich particles and their remnants in atherosclerotic cardiovascular disease [2, 29].

The aim of this study is to describe the clinical, biochemical and genetic characteristics of FHTG, in order to identify peculiarities which, distinguish this disease from other forms of HTG. A secondary aim is to analyze differences in the circulating levels of regulatory proteins related to triglyceride homeostasis between 171 study subjects, (composed of 84 FHTG individuals and 85 CHTG individuals) and 73 control individuals. These regulatory proteins include three members of the secreted Fibroblast Growth Factor (FGF) family of proteins namely fibroblast growth factor 19 (FGF-19) [30], fibroblast growth factor 21(FGF-21) [31] and fibroblast growth factor 23 [32]. FGF-21 regulates hepatocyte and adipocyte metabolism, FGF-19 is a regulator of bile acid synthesis (increased bile acid synthesis is seen in hypertriglyceridemia), and FGF-23 has recently been implicated in cardiovascular risk. The measurement of angiopoietin-like proteins 3 and 4 (ANGPTL-3 and ANGPTL-4) was also carried out. These proteins exhibit functional roles in lipid metabolism and inflammation. They are regulators of lipoprotein and glucose metabolism. ANGPTL-3 function is linked to the inhibition of lipoprotein lipase (LPL) activity, the principal enzyme that hydrolyzes triglycerides [33] and to the activation of lipolysis in adipocytes, a process resulting in FFA and glycerol release into the circulation [33]. ANGPTL-3 is a predictor of peripheral artery disease, associated with endothelial dysfunction, and is thought to stimulate the proliferation of prothrombotic and proinflammatory cells, exacerbating atherosclerosis [33]. The inhibition of ANGPTL3 is a promising pharmacological target for the prevention of coronary artery disease in hypertriglyceridemia especially in persons with FHTG in which the control of triglycerides levels is a challenge. The GalNAc-conjugated ANGPTL3 ASO, targets ANGPTL3 synthesis in the liver, reducing atherogenic lipoproteins. ANGPTL4 is regulated by different nuclear receptors, it is induced by fasting and is produced in liver, muscle, liver and adipose tissue. ANGPTL4 has an important role in fat metabolism and adipose tissue differentiation. The regulation of LPL by ANGPTL4 is important for protection from the proinflammatory effects of lipid accumulation [33].

In addition, the measurement of liver-type fatty acid-binding protein (FABP1/L-FABP), was also included as this protein is associated with dyslipidemia (elevated plasma triglycerides) and non-alcoholic fatty liver disease (NAFLD), a condition commonly seen in FHTG subjects [34]. Finally, the measurement of apolipoproteins including apo A-II and apolipoprotein CIII (apo C-III) was carried out in all subjects [35].

The measurement of these regulatory proteins will permit the detection of a set of variables that better identify FHTG cases.

## Material and methods

### Study participants

A total of 1421 subjects were stratified into 3 groups according to triglyceride levels. Two of the groups were prospectively enrolled over a twelve-month period (January to December 2019) from the lipid clinic of the Instituto Nacional de Ciencias Médicas y Nutrición Salvador Zubirán (INCMNSZ). The initial consultation included a medical history, physical examination and fasting blood samples for biochemical measurements (lipid profile, glucose, thyroid function, renal and hepatic function tests) and DNA extraction. All subjects signed informed consent on entering the study. The study protocol conformed to the ethical guidelines of the 1975 Declaration of Helsinki and was approved by the Research and Ethics Committee of the hospital. The 3 groups of subjects were: 1) eighty-four unrelated cases with FHTG defined as TG levels ≥1000 mg/dL (≥ 11.3 mmol/l) on at least one occasion, with apo B levels <90th population percentile for age and gender and a 5:1 TG/cholesterol ratio, after excluding other causes of HTG [22]. The study subject had to have at least one first-degree relative with the same lipid patterns. 2) a group pf 728 subjects with CHTG defined as TG levels ≥150 mg/dL but < 600 mg/dL (> 2.26 but < 6.78mmil/l), low-density lipoprotein cholesterol (LDL-C) levels < 190 mg/dL (< 4.921 mmol/l) estimated by Martin and Sampson formulas [36, 37], and 3) 609 individuals from the UNAM-INCMNSZ Diabetes Study, who participated in the SIGMA T2D Consortium [38] and met the eligibility requirements for normotriglyceridemic controls. These subjects had normal fasting TG levels < 150 mg/dL (1.7 mmol/l) and normal HDL-C levels (40-60 mg/dL, 1.04–1.56 mmol/l). For the analysis of the metabolic profile, all 84 FHTG were included plus subgroups of 85 CHTG and 73 control individuals for whom serum samples were available. Subgroups were matched by gender, body mass index (BMI) and waist circumference to permit comparisons among groups. However, adjustment for age was necessary.

Exclusion criteria included pregnancy, alcoholism (defined as the consumption of at least 10 alcoholic beverages within a 2-month period), uremia (serum creatinine > 176.8 μmol/l), nephrotic syndrome, hemodialysis, severe liver failure, lipid-lowering drugs, HIV infection, acute stress events in the 6 weeks before entering the study, untreated thyroid disease or other autoimmune diseases and an FCS score ≥ 10 [8].

### Anthropometric measurements

Anthropometric measurements included weight and height estimations with calibrated scales and stadimeters. Waist-circumference was measured at the mid-point between the lower border of the costal margin and the iliac crest during exhalation. Arterial hypertension was defined as a systolic blood pressure (SBP) ≥ 140 mmHg, diastolic blood pressure (DBP) ≥90 mmHg or self-reported use of antihypertensive medications [39]. Smoking status was recorded and individuals were classified as current smokers (smoking at least one cigarette in the previous month), former smokers (smoking the last cigarette more than 6 months ago) or never smokers.

### Biochemical measurements

All measurements were performed after a 12-h fast. Samples were taken by venous puncture and distributed into tubes BD Vacutainer® RST and were immediately stored on ice until clot was formed and centrifuged at 1500 rpm for 15 min. Glucose, total cholesterol and HDL-C measurements were performed using Synchron CX Delta (Beckman Coulter) colorimetric enzymatic methods and apo B measurements were performed by nephelometry (Beckman Coulter). The homeostasis model assessment for insulin resistance (HOMA2-IR) was calculated using the HOMA2 calculator released by the Diabetes Trials Unit, University of Oxford, available at: http://www.dtu.ox.ac.uk/homacalculator/index.php. Leptin and adiponectin were measured using ELISA (Millipore microplates). FGF-21, FGF-19, FGF-23, ANGPTL3, 4, apo C-III were measured by MILLIPLEX® MAP, according to the manufacturer’s protocols.

### DNA purification and SNP genotyping

Genomic DNA was extracted from whole blood using the QIAmp 96 DNA Blood Kit. Purity and concentration were obtained with a NanoDrop ND 1000. DNA samples were genotyped at LGC Genomics (Beverly, MA, USA) using KASP genotyping technology, which is a competitive allele-specific PCR that enables highly accurate bi-allelic scoring of SNPs. On a first PCR round, it uses three assay-specific non-labelled primers: one common reverse primer plus two allele-specific forward primers. On a second PCR round, a fluorescent signal is generated using fluor-labelled FRET cassettes. SNPs with a call rate < 97% were considered technical failures and were automatically removed before further quality control.

### Variants selection criteria and quality control procedure

Two sets of markers were genotyped. The first one was selected to explore the *polygenic component* of TG levels and included 53 common tag SNPs, one per gene, previously associated to TG levels, as reported by Willer et al. and Weissglass-Volkov *et.*al [19, 40]. The second set of markers was selected to explore the *monogenic component* and included 37 genetic variants located within the five main genes associated with FCS (i.e. *LPL*, *APOC2*, *APOA5*, *LMF1* and *GPIHBP1*) and consistently found in FCS and FHTG cases [10–14] (Supplementary Tables 1 and 2).

Specifically, for the polygenic component set, quality control included the exclusion of samples with ≥10% missing data within the full dataset or whose call rate between cases and controls was statistically different (*P <* 0.00001). In addition, variants with ≥5% missing data and monomorphic variants were also removed. All SNPs were tested for Hardy-Weinberg equilibrium (*P* < 0.000001). SNPs that failed to pass the test were excluded for further analyses. In turn, except for case-control call rate difference, minor allele frequency and Hardy-Weinberg equilibrium filters, quality control of the monogenic component set included the same criteria. After quality control, whole dataset included 1421 individuals (84 FHTG cases, 728 CHTG patients and 609 normolipidemic controls), 38 SNPs from the polygenic component set (1 SNP failed HWE test and 14 SNPs were monomorphic) and 37 variants from the monogenic component set.

#### Statistical analyses

The distribution of continuous variables was explored using the Kolmogorov-Smirnov test. Data are presented as median and interquartile range. Categorical variables are reported as frequencies and percentages. Continuous variables were compared among FHTG, CHTG and control subjects using the Kruskall-Wallis test, with the Dunn Test post hoc analysis. The frequency distribution of the categorical variables was compared using chi-squared test. In order to control for population structure, an ancestry index was calculated through principal components analysis using a validated set of 32 ancestry informative markers for Latino populations [41] via the EIGENSTRAT software [42]. The top 2 principal components (PC) were used as covariates in further analyses.

The genetic association between variants of the polygenic component set with FHTG and CHTG was assessed using logistic regression models. For the first analysis FHTG individuals were the cases and normotriglyceridemic subjects were the controls. Similarly, for the second analysis, CHTG were the cases and normotriglyceridemic subjects were the controls. Models were adjusted for age, age^2^, sex, BMI, T2D status, and the top 2 PCs to account for ancestry. The effect of each variant was assessed separately. Since all variants have already been proved to be associated with TG levels, nominal *p*-values without correction for multiple comparisons are reported. Associations with *P* < 0.05 were considered statistically significant. For the variants of the monogenic component set, only allele frequencies among FHTG individuals are reported.

Finally, the TG variance explained specifically by the polygenic set of genetic variants was calculated, as described by Purcell et al., 2007 [43]. Briefly, for each individual, a genetic risk score (GRS) using PLINK software (i.e. number of score alleles weighted by the previously reported beta coefficients in the literature) was estimated. Scores were additive across SNPs. No scores were imputed for missing genotypes. Next, the TG variance explained by the GRS as the difference in the r^2^ from a linear regression model including the score and covariates and a linear regression model excluding the score was calculated. Defined covariates were sex, age, age^2^, BMI, T2D status, ancestry and the number of non-missing genotypes used to calculate each GRS. A bootstrapped 97% confidence interval for R-squared was generated based on 10,000 replications. PLINK software and R were used for all genetics analyses.

In addition, an analysis of proinflammatory parameters was also carried out to see if they were useful to distinguish between FHTG and CHTG. To evaluate the contribution of additional metabolic parameters to routine clinical measurements support vector machines (SVM) models with the e1071 R package were constructed, optimizing hyperparameters using cross-validation tuning to assess models comprising the identified pathophysiological components. To estimate performance of SVM models comparing the addition of metabolites, confusion matrices, area under the receiver operating characteristic (ROC) curves (c-statistic) and selected the cut-off value with optimal balance between sensitivity and specificity using the Optimal Cutpoints R package computing the Youden Index were estimated to evaluate discrimination between FHTG, CHTG and controls [44] . The models were adjusted for age, gender and BMI, apo B and then compared when each additional metabolite was included; thresholds for individual variables were evaluated and clinically useful models for the detection of FHTG compared to CHTG and control subjects using sequential logistic regression models were constructed, assessing model selection using ROC curves and the Bayesian Information Criterion (BIC); the better model had the better AUC of ROC and the lowest BIC. Stata v13.0, SPSS v25.0 and R v3.6.1 were used to perform analyses [45].

## Results

### Clinical and biochemical characteristics of all study subjects

Table 1 shows the characteristics of the study group, which included 1421 individuals (84 FHTG cases, 728 CHTG individuals, and 609 normotriglyceridemic controls. FHTG subjects were younger compared to the CHTG group, and the FHTG group had a lower proportion of female participants. Not a single FHTG subject met the diagnostic scoring system criteria for FCS (≥10) and none had eruptive xanthomas [8]. The prevalence of T2D was higher in FHTG subjects and 20% had a history of acute pancreatitis. FHTG subjects had lower HDL-C, non-HDL-C and apo B levels (*P* < 0.001) and higher concentrations of insulin, and HOMA IR (*P* < 0.001). Supplementary Table 5 shows the data of the patients divided by T2D status.

**Table 1:** Clinical and biochemical characteristics of study subjects

Variable	Normal Triglycerides	CHTG	FHTG	***P*** value ALL	***P*** value* FHTG vs CHTG
Sample size	609	728	84		
Sex (% females) ^a^	69.2	51.1	24.4	**< 0.001**	**< 0.001**
Age (years)	54 [48–63]	53 [48–60]	43 [37–47.8]	**< 0.001**	**< 0.001**
BMI (kg/m^2^)	27 [24.7–30]^b^	28.6 [25.9–31.6]	28.9 [26.1–32.1]	**< 0.001**	**0.718**
Waist circumference (cm)	90.8 [84.5–98]^b^	95.4 [89.5–103]	96 [88–100]	**< 0.001**	**0.581**
Triglycerides (mg/ dL)	113 [88.57–130]	270 [228–331.25]	916.46 [293.24–1435.44]^c^	**< 0.001**	**< 0.001**
Cholesterol (mg/ dL)	195 [168.52–218.31]	217 [190–243]	191.02 [163–250.87]^c^	**< 0.001**	**0.027**
HDL-C (mg/ dL)	47 [42–55]	38 [32–44]	30.64 [24.89–37.34]	**< 0.001**	**< 0.001**
Non- HDL-C (mg/ dL)	145 [121–168]	176 [154–201]	159 [130–216] ^c^	**< 0.001**	**0.288**
Apo B (mg/ dL)	103 [86–118]	126 [108–143]	91.8 [82–97.6]	**< 0.001**	**< 0.001**
Glucose (mg/ dL)	90 [83–108]^b^	94 [85–148.25]	99 [90.5–119]	**< 0.001**	**0.361**
Insulin (μU/L)	8.3 [5.6–12]^b^	10.35 [7.57–15.7]	13.3 [7.9–18.8]	**< 0.001**	**0.036**
HOMA2 B%	106.2 [81.3–139.4]	119.85 [92.6–159.7]^d^	102.2 [65.8–154.4]	**< 0.001**	**0.002**
HOMA IR	1.06[0.72–1.56]^b^	1.37 [1.0–2.06]	1.79 [1.17–2.28]	**< 0.001**	**0.004**
T2D (%)^a^	31.7	39.8	25.6	**< 0.001**	**0.010**
AP (%)	–	–	20%	**< 0.001**	**< 0.001**

Median [25th percentile-75th percentile] or percentages are shown. AP: Acute pancreatitis

^a^ Chi square/Fisher exact test

Kruskall Wallis test for metabolic traits adjusted for age, BMI, triglycerides levels and sex;

^b^ post hoc *P* < 0.01 by Dunn test: Normal triglycerides subjects different among the other groups

^c^ post hoc *P* < 0.01 by Dunn test: FHTG subjects different only from CHTG subjects

^d^ post hoc *P* < 0.01 by Dunn test: CHTG subjects different among the other groups

*U-Mann Whitney test

### Pro-atherogenic profile and endocrine regulatory proteins closely related to triglyceride homeostasis

Table 2 shows the comparison between all FHTG cases and a subsample of CHTG and controls. There were no significant differences in BMI, gender and waist circumference between FHTG and CHTG groups; however, the FHTG group was younger (*P* = 0.005). Normal triglyceridemic individuals were younger with lower BMI, so all the comparisons were adjusted for age, gender, BMI and triglycerides levels. FHTG subjects showed lower levels of apo B and leptin levels and significantly higher levels of FGF-21, ANGPTL3, ANGPTL4 compared to CHTG and controls (*P* < 0.001). FHTG individuals showed higher levels of apo A-II compared to CHTG subjects (*P* < 0.001).

**Table 2:** Atherogenic profile of study subjects

	Normal Triglycerides ***n*** = 73	CHTG ***n*** = 85	FHTG ***n*** = 84	***P value*** ***all***	P value* FHTG vs ***CHTG***
Age (years)	49 [45–54]	50 [37–55]	43 [37–47] ^b^	**0.002**	**0.001**
BMI (kg/m^2^)	26.4 [24.2–28.7] ^a^	28.7 [25.9–31.1]	28.9 [26.1–32.1]	**0.008**	**0.813**
Waist (cm)	90 [84–98] ^a^	94 [88–104]	96 [88–100]	**0.004**	**0.800**
Leptin (μg/L)	5.6 [2.3–13.6]	8.7 [7.2–28]	5.7 [2.6–8.7]	**< 0.001**	**< 0.001**
Adiponectin (μg/L)	11.4 [8.8–14.6] ^a^	6.8 [4.3–10.4]	5.9 [4.5–8.4]	**< 0.001**	**0.146**
Apo B (mg/dL)	103 [86–114]	110 [109–117]	91.8 [82–97.6]	**< 0.001**	**< 0.001**
Apo A-II (mg/dL)	1894 [996–2858]	426.5 [401.4–456.5]	702 [497–1062]	**< 0.001**	**< 0.001**
Apo C-III (mg/dL)	10.7 [7.3–13.4]	30.68 [24.5–38]	22.8 [13.6–37]	**< 0.001**	**0.231**
ANGPTL3 (ng/mL)	9 [6.6–11.2]	18.8 [13.6–22]	108 [10.8–372]	**< 0.001**	**< 0.001**
ANGPTL4 (ng/mL)	50.5 [21.5–145.9]	180.9 [151.9–225.3]	384 [239–483]	**< 0.001**	**< 0.001**
FGF-19 (pg/mL)	15 [10–81]	15 [7–18]	77 [10–156] ^b^	**0.052**	**0.071**
FGF-21 (pg/mL)	55.5 [32–89]	12.5 [2–22]	168 [66–316]	**< 0.001**	**< 0.001**
FGF-23 (pg/mL)	25 [18–38]	17.5 [16–22]	33[22–50]	**0.127**	**0.417**
FABP1 (ng/mL)	37 [2.15–302.1] ^a^	15.5 [13.6–20.8]	13 [1–45]	**0.010**	**0.056**

Median [25th percentile-75th percentile] or percentages are shown

Kruskall Wallis test for metabolic traits adjusted for age, BMI, triglycerides levels and sex;

^a^ post hoc *P* < 0.01 by Dunn test: Normal triglycerides subjects different among the other groups

^b^ post hoc *P* < 0.01 by Dunn test: FHTG subjects different only from CHTG subjects

^c^ post hoc *P* < 0.01 by Dunn test: CHTG subjects different among the other groups

*U Mann-Whitney test

All the exploratory biomarkers were useful to distinguish between FHTG, CHTG and controls. The most informative metabolites were FGF-21, apo A-II and ANGPTL3. The ANGPTL3 threshold was > 198 ng/mL (AUC 0.819, 95% CI 0.761–0.877) with a sensitivity of 0.95 (95% CI 0.91–0.98) and a specificity of 0.50 (95% CI 39–61) to detect FHTG. For FGF-21, the threshold was > 87 pg/mL (AUC 0.788, 95% CI 0.728–0.848), with a sensitivity of 0.71 (95% CI 0.61–0-.80) and a specificity of 0.75 (95% CI 0.68–0.82). Finally, for apo A-II the threshold was > 0.472 mg/dL (AUC 0.878, 95%CI 0.824–0.932), with a sensitivity of 0.86 (95%CI 0.76–0.92) and a specificity of 0.79 (95%CI 0.68–0.88).

An SVM model was constructed to discriminate FHTG from CHTG and control subjects which included gender, age, BMI and apolipoprotein B levels. The model had an AUC of 0.852 (95%CI 0.786–0.904, 96.15% sensitivity, 79.61 specificity). The inclusion of FGF-21, ANGPTL3 and apo A-II together with clinical variables showed the best performance (AUC 0.948, 95%CI 0.901–0.970, 98.5% sensitivity, 92.2% specificity, *P* < 0.001) of all the explored models (Table 3). If T2D subjects were excluded, the model showed an AUC 0.969, (95%CI 0.938–0.999).

**Table 3:** Performance of individual and combined factors in predicting FHTG compared to CHTG using logistic regression analyses. *Model adjusted for age

Variable	AUC (95% CI)	Cut-off value	BIC	Sensitivity (95%CI)	Specificity (95%CI)	PPV (95%CI)	NPV (95%CI)
Age (years)	0.633 (0.539–0.727)	< 45.0	217.80	72.6(61.8–81.8)	67.0 (56.0–76.9)	68.8 (58.0–78.1)^f^	66.3 (55.4–76.2)
Apo B (mg/dL)	0.853(0.787–0.918)	< 102.0	145.29	92.8 (85.1–97.3)	73.3 (61.8–82.9)	81.8 (68.5–87.4)	57.6 (46.4–76.5)
Apo A-II (mg/dL)	0.878(0.824–0.932)	> 0.472	143.22	85.7 (76.4–92.4)	78.8 (68.6–86.9)	77.0 (65.2–83.8)	60.9 (50.3–74.9)
ANGPTL3 (ng/mL)	0.530(0.433–0.628)	> 214.7	199.56	48.8 (37.7–60.0)	91.5 (82.5–96.8)	87.2 (74.8–91.5)	60.2 (49.0–81.0)
FGF-21(pg/mL)	0.804(0.737–0.872)	> 27.0	204.70	96.4 (89.9–99.2)	57.6 (46.4–68.3)	60.0 (28.7–81.9)	47.9 (15.7–88.5)
Sex + Apo B < 102 mg/ dL	0.858 (0.799–0.917)	> 0.66	149.91	90.5 (82.1 98.9)	73.2 (61.4–85.0)	80.0 (69.9–90.1)	86.7 (75.8 97.6)
Apo B < 102 mg/dL + apo A-II > 0.472 mg/dL	0.917 (0.874–0.960)	> 0.94	114.62	76.2 (65.6–84.8)	92.9 (84.3–97.7)	92.7 (83.9–95.7)	76.7 (66.3–91.3)
Apo B < 102 mg/dL + apo A-II > 0.472 mg/dL + Sex	0.934 (0.894–0.975)	> 0.49	113.6	95.2 (88.2–98.7)	78.9 (67.6–87.7)	84.2(74.8–95.2)	93.3 (84.0–96.4)
Apo B < 102 mg/dL + apo A-II > 0.472 mg/dL + Sex*	0.944 (0.908–0.981)	> 0.64	114.09	89.3 (80.6–95.0)	85.9 (75.6–93.0)	88.2 (79.2–94.4)	87.1 (77.2–93.7)
Apo B < 102 mg/dL + apo AII > 0.472 mg/dL + Sex + FGF-21 > 27 pg/mL, ANGPTL3 > 214 ng/mL*	0.969 (0.920–0.999)	> 0.45	107.88	98.9 (94.5–99.9)	92.5(78–92)	89.3 (80.7–99.7)	98.7 (92.3–99.2)

*PPV* Positive predictive value; *NPV* Negative predictive value; *AUC* Area under curve; *BIC* Bayesian information Crite

In the SVM models, none of the individual clinical variables were as strong as the added predictors and the combination of variables was decidedly superior. Finally, a clinical model was developed to easily identify FHTG using logistic regression analyses. A model comprised of, FGF-21 > 27.0 pg/mL, apo A-II > 0.472 mg/dL, and apo B < 102 mg/dL, showed the best performance to differentiate FHTG from CHTG; the addition of ANGPTL3 > 214 ng/mL improved model performance.

### Role of the common polygenic TG component with FHTG

The role of 53 common SNPs (previously associated with TG levels and CHTG) included in the polygenic component set, were assessed [41–43]. Only 38 out of the 53 SNPs were evaluated given that 14 were shown to be monomorphic in the Mexican population (Suppl. Table 1). Only eight variants reached statistical significance for an association with FHTG (Suppl. Table 2): *APOA5/BUD13* (rs964184, *P* = 2.54 × 10^− 07^), *LIPC* (rs1532085, *P* = 0.0055), *INSR* (rs7248104, *P* = 0.0071), *GCKR* (rs1260326, *P* = 0.0155), *LRPAP1* (rs6831256, *P* = 0.0247), *PLTP* (rs6065906, *P* = 0.0304), *CYP26A1* (rs2068888, *P* = 0.0366) and *MLXIPL* (rs2286276, *P* = 0.0375) (Suppl. Table 2). In contrast, seven variants were associated with CHTG: *APOA5/BUD13* (rs964184, *P* = 7.7 × 10^− 12^), *GCKR* (rs1260326, *P* = 0.0046), *LRP1* (rs11613352, *P* = 0.0069), *JMJD1C* (rs10761731, *P* = 0.0177), *CILP2* (rs10401969, *P* = 0.0246), *CTF1* (rs11649653, *P* = 0.0457) and *LPL* (rs12678919, *P* = 0.0577) (Suppl. Table 2). However, when a genetic risk score was developed using the complete set of 38 known TG associated variants, it explained only 1.78% [0.45–44.47] of the variance in TG levels in the FHTG sample, and 6.73% [0.47–9.56] of the variance in TG levels in the CHTG sample (Table 4). A similar pattern of allele frequencies was seen between CHTG and FHTG groups, except for, except for *APOA5/BUD13* rs964184, which showed higher frequency in FHTG compared to CHTG (Suppl. Figure 1).

**Table 4:** The explained variance in TG levels using a polygenic risk score in FHTG and CHTG

Group	Explained variance (%) [IC 0.025–0.975]	***P***
FHTG	1.78 [0.45–4.47]	< 2.2 × 10^−16^
CHTG	6.73 [0.47–9.56]	

IC from bootstrapped R-squared of models performing linear regression of TG levels on polygenic scores. The polygenic risk score was computed using the complete set of 38 known TG associated variants. *P* value of the comparison between the variance explained in FHTG versus CHTG

### Role of the rare monogenic TG component with FHTG

Regarding the monogenic component set, 37 genetic variants previously reported as associated with FCS and FHTG were analyzed (Suppl. Table 3). Only 5 of the 37 variants were present in the FHTG patients (*LPL*, p.Val227Ala, *GPIHBP1* rs72691625, *LMF1* rs4984948, *LMF1* rs35168378 and *LMF1* rs143076454). However, except for *LPL*, p. Val227Ala, the other 4 variants were also present in the normoglycemic and CHTG subgroups, suggesting that they do not drive the FHTG phenotype. Furthermore, 2 additional variants were found in the normoglycemic subgroup but not in the FHTG subgroup (Suppl. Table 4). Finally, the same pattern of allele frequencies was seen between CHTG and FHTG groups (Suppl. Figure 1).

## Discussion

This study demonstrates that FHTG has clinical and biochemical characteristics that distinguish it from other forms of common mild-to-moderate hypertriglyceridemia (CHTG). After controlling for confounding variables, FHTG cases have lower HOMAB%, HDL-C, apo B, and leptin levels and higher FGF-21, ANGPTL3 and ANGPTL4 levels than CHTG subjects. Only apo B, FGF-21, ANGPLT3 and apo A-II concentrations, along with gender, age and BMI were independent components of a model to detect FHTG compared with CHTG and normotriglyceridemic controls (AUC 0.948, 95%CI 0.901–0.970, 98.5% sensitivity, 92.2% specificity, *P* < 0.001).

**Fig. 1:**
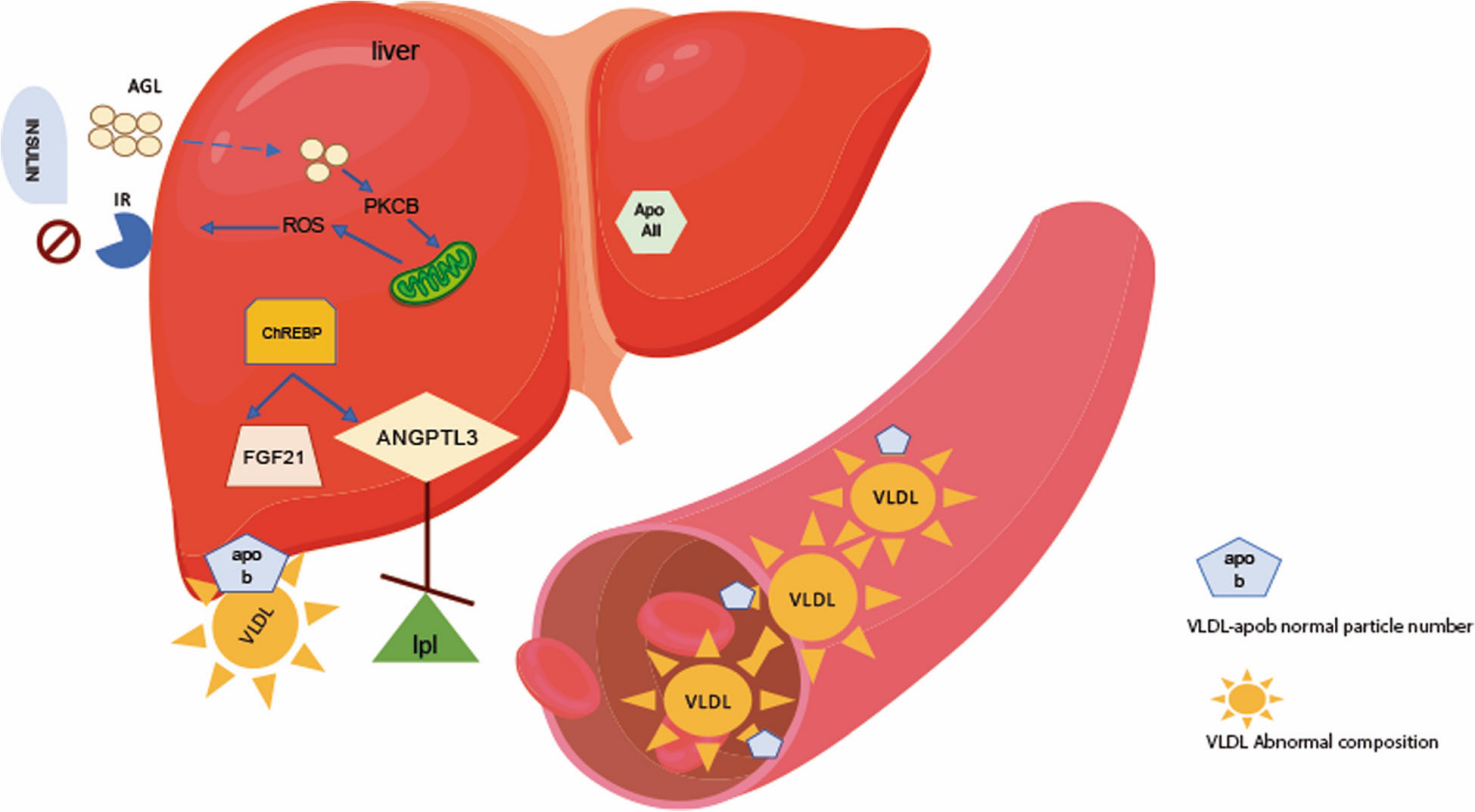
Proposed physiopathological mechanisms that may contributed to the genesis of familial hypertriglyceridemia. Inhibition of LPL orchestrated by FGF21 and ANGPTL3, both of which are regulated by CHREBP. This results in alterations in the composition of VLDL particles with numbers remaining unchanged

The main pathophysiological mechanism of hypertriglyceridemia in FHTG is abnormal VLDL composition without alteration in VLDL particle number [21]. As a result, plasma apo B levels in FHTG subjects are normal or low. High TG levels in FHTG subjects are not determined by hepatic VLDL-apo B overproduction, it is likely to be a result of decreased TG clearance (Fig. 1) [21].

*FGF-21* and *ANGPTL3,* mRNA and protein levels are regulated by the carbohydrate response element-binding protein (ChREBP) [46, 47]. Therefore, ChREBP, a transcriptional activator of glycolytic and lipogenic genes may play a role in the development of hypertriglyceridemia in FHTG by regulating the secretion of those molecules (Fig. 1) altering the VLDL-particle composition without changing their numbers. Interestingly, there was a stronger association of *ChREBP* gene with FHTG than with CHTG (*P* = 0.037 and 0.092, respectively, Suppl. Table 2).

In humans, serum FGF-21 correlates with de novo lipogenic rates measured by stable isotopic tracers [31], contributing to increased hepatic triglycerides synthesis. Lee Y et al. showed that FGF-21 concentrations positively correlate with TG, insulin and HOMA-IR – [48, 49], and Akyildiz Zl et al. showed that TG might play an important role in predicting FGF21 concentrations [50]. Endoplasmic reticulum (ER) stress is another factor that may increase FGF21 concentrations, it is well known that FHTG subjects have high postprandial lipemia that may lead to atherosclerosis and endothelial dysfunction with ER stress [51]. Insulin resistance and the affluence of fatty acids towards the liver contributes to the inflammatory process seen in FHTG.

Furthermore, the higher concentration of angiopoietin-like 3 may cause decreased triglycerides clearance [52]. ANGPTL-3 is a major inhibitor of lipoprotein lipase (LPL) activity, the principal triglycerides hydrolyzing protein [53, 54]. Recent evidence has confirmed the prominent role that inhibitors of ANGPTL3 have in the removal of triglycerides-rich particles, LDL-C and apo B levels, probably due to an increased clearance of apo B containing lipoproteins and their remnants [33]. Monoclonal antibodies and antisense oligonucleotides that target ANGPTL3, and 4 are potentially an efficient therapeutic strategy for cardiovascular risk reduction in hypertriglyceridemia, especially in patients with FHTG [55, 56]. The results suggest that this molecule may be involved in the pathogenesis of FHTG and its measurement may be helpful for diagnosis (Fig. 1). A recent publication by Chen YQ et al. showed how during feeding, ANGPTL8 forms a circulating complex with ANGPTL3 that increases its ability to inhibit LPL, thus minimizing FA uptake into skeletal muscle [57].

**Fig. 2:**
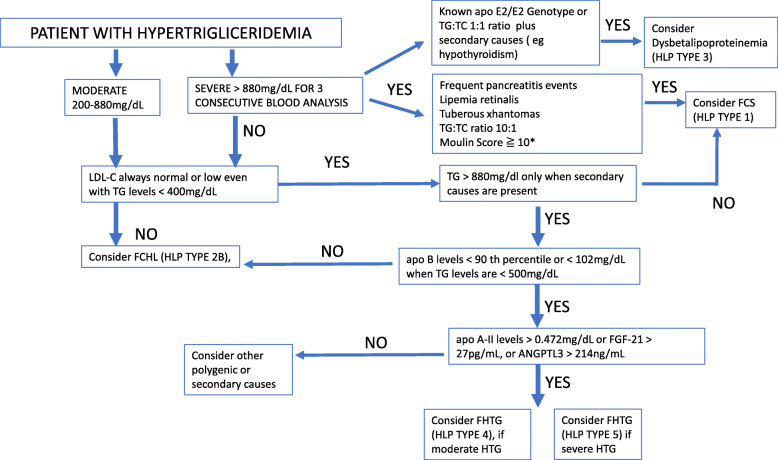
apo B: apolipoprotein B, apo A-II:apolipoprotein A-II; FCHL: Familial Combined Hyperlipidemia, FCS: Familial Chylomicronemia Syndrome; FHTG: Familal Hypertriglyceridemia; HLP: hyperlipidemia, LDL-C: low-density lipoprotein cholesterol; TC: total cholesterol; TG: triglycerides; * see ref. [8]

Therefore, a model using a combination of apo B, FGF-21, ANGPTL3 and apo A-II levels is proposed in order to improve identification of FHTG subjects from the wide constellation of hypertriglyceridemic phenotypes (Figs. 1 and 2) This model showed an excellent performance in this cohort of patients.

Regarding the genetic components, 8 out of 44 common variants were found to be associated with FHTG, and only two were also associated with CHTG (*APOA5/BUD13*, rs964184 and *GCKR,* rs1260326). The set of variants associated with FHTG but not with CHTG included *LIPC*, rs1532085, *INSR*, rs7248104, *LRPAP1*, rs6831256, *PLTP*, rs6065906 *CYP26A1*, rs2068888 and *MLXIPL*, rs2286276.

Notably, none of the main 5 genes associated with FCS explained the FHTG phenotype in these patients. Despite the fact that some of the FHTG patients were carriers of previously reported variants, they were also present in the normoglycemic subgroup. Only p. Val227Ala within the *LPL* gene was present in FHTG but not in normotriglyceridemic participants.

A primary cause of FCS is the recessive inheritance of rare variants in certain genes and that FHTG patients may be compound heterozygotes (i.e. heterozygous carriers of more than 1 rare variant): none of the FHTG patients were homozygous for any of the studied rare variants. Furthermore, compound heterozygote (rs35168378 + rs143076454) patients were identified in both normotriglyceridemic and FHTG patients.

The results show that neither the previously known set of common variants, nor a set of monogenic variants, explain a significant proportion of the genetic contribution to FHTG in Mexican subjects. Over the past years, many GWAS of dyslipidemias have been performed, but only a small percentage of the total variance in phenotypes has been explained by the genetic component. Thus, even though the current results are insufficient to accurately assess the likelihood of developing hypertriglyceridemia, the findings may be helpful in directing studies towards new disease mechanisms and targets for prevention and therapy. This highlights the need for the identification of additional genetic components of FHTG through free-hypothesis approaches. Previously, we have reported that the Mexican population has genetic variants which are unique to this and other Native American-derived populations. Such variants have higher effect sizes, explain a greater percentage of the variance and its corresponding genes and pathways may represent new potential therapeutic targets [58–60]. The results highlight the need for a more extensive genetic characterization of FHTG through whole exome/genome sequencing to identify additional ethnic-specific gene variants.

### Study strengths and limitations

This is the first attempt for the complete description and characterization (clinical, biochemical, genetic) of the FHTG phenotype, which is frequently confused with other HTG phenotypes.

We propose a whole genome sequencing approach given that the Mexican population shows genetic variation that occurs at a very low frequency or that is absent among other populations. Moreover, such variation may have a higher effect size compared to other populations. Although most evidence has been provided for the genetics of type 2 diabetes the same may presumably happen with other metabolic diseases, such as hypertriglyceridemia [58, 59]. Finally, we recognize as a limitation of the study, the lack of information on the contribution of heterozygous forms of causal variants of FCS, although there were a few individuals who were heterozygous carriers of FCS mutations. In addition, the number of subjects included in the analysis of pro-atherogenic profile and endocrine regulatory proteins was small. Furthermore, it is necessary to authenticate these findings in other populations and with a greater number of individuals, to improve the external validity of the results. A family study for all the probands in the CHTG group was not available so we cannot definitively confirm that these individuals belonged to the FCHL phenotype, but the lipid profile is characteristic of this disease, especially when FCHL patient have a hypertriglyceridemia phenotype plus a secondary cause [17].

## Conclusions

We propose that FHTG is a unique entity having a distinct clinical, biochemical and genetic profile that distinguishes it from other forms of HTG (Fig. 2). It is characterized by higher concentrations of insulin, FGF-21, ANGPTL3, apo A-II and lower levels of apo B. Furthermore, neither the previously known set of common variants, nor a set of monogenic variants, explain a significant proportion of the genetic contribution to FHTG in Mexican subjects. This study is an important step in the study of hypertriglyceridemia confirming the presence of subtle biochemical and genetic differences associated with different HTG phenotypes.

## Supplementary Information

**Additional file 1.**

**Additional file 2.**

## Data Availability

The datasets used and/or analysed during the current study are available from the corresponding author on reasonable request.

## Abbreviations

ANGPTLs: Angiopoietin-like proteins
Apo AII: Apolipoprotein A-II
Apo B: Apolipoprotein B
BIC: Bayesian Information Criterion
BMI: Body mass index
CHTG: Common mild-to-moderate hypertriglyceridemia
FABP1/L-FABP: Liver-type fatty acid-binding protein
FCHL: Familial combined hyperlipidemia
FCS: Familial chylomicronemia syndrome
FGFs: Fibroblast growth factor proteins
FHTG: Familial hypertriglyceridemia
GPIHBP1: Glycosylphosphatidylinositol-anchored high-density lipoprotein-binding protein 1
GRS: Genetic risk score
GWAS: Genome-wide association studies
HDL-C: High-density lipoprotein cholesterol
HLP: Hyperlipidemia
HTG: Hypertriglyceridemia
LDL-C: Low-density lipoprotein cholesterol
LMF1: Lipase maturation factor 1
LPL: Lipoprotein lipase
NAFLD: Non-alcoholic fatty liver disease
ROC: Receiver operating characteristic
SNPs: Single nucleotide polymorphisms
SVM: Upport vector machines
TG: Triglycerides
T2D: Type 2 diabetes
VLDL: Very low-density lipoprotein cholesterol
