# Supplementary material for: Familial hypertriglyceridemia: an entity with distinguishable features from other causes of hypertriglyceridemia
Source: Lipids Health Dis. 2021 Feb 15;20:14. doi: 10.1186/s12944-021-01436-6 (PMC7885394; doi:10.1186/s12944-021-01436-6)
Supplement: Supplementary file 1 — Additional file 1. [file 12944_2021_1436_MOESM1_ESM.docx]

**SUPPLEMENTARY TABLES**

**Supplementary Table 1. Polygenic component set of genetic variants analyzed in this study.**

| **SNP** | **CHR** | **BP** | **ALLELES** | **GENE** | **PREDICTED FUNCTION** | ***f* This study** | ***f* EUR** | ***f* ASN** | ***f* AFR** |
| --- | --- | --- | --- | --- | --- | --- | --- | --- | --- |
| rs10401969 | 19 | 19407718 | T/C | *SUGP1* | intronic | C:0.03521 | C:0.065 | C:0.090 | C:0.175 |
| rs10761731 | 10 | 65027610 | A/T | *JMJD1C* | intronic | T:0.1881 | T:0.394 | T:0.353 | T:0.279 |
| rs10889337 | 1 | 62980607 | G/A | *DOCK7* | intronic | A:0.4655 | A:0.337 | A:0.209 | A:0.595 |
| rs1121980 | 16 | 53809247 | G/A | *FTO* | intronic | A:0.2126 | A:0.459 | A:0.204 | A:0.494 |
| rs11613352 | 12 | 57792580 | C/T | *R3HDM2* | intronic | T:0.5657 | T:0.203 | T:0.103 | T:0.049 |
| rs11649653 | 16 | 30918487 | C/G | *CTF2P* | non-coding intronic | G:0.4977 | G:0.408 | G:0.897 | G:0.023 |
| rs1260326 | 2 | 27730940 | T/C | *GCKR* | coding nonsyn | G:0.7066 | C:0.574 | C:0.454 | C:0.845 |
| rs12678919 | 8 | 19844222 | A/G |  | intergenic | G:0.02871 | G:0.113 | G:0.126 | G:0.078 |
| rs12748152 | 1 | 27138393 | C/T |  | upstream gene | T:0.01636 | T:0.065 | T:0.023 | T:0.009 |
| rs1532085 | 15 | 58683366 | A/G | *ALDH1A2* | intronic | G:0.6643 | G:0.622 | G:0.459 | G:0.466 |
| rs17145738 | 7 | 72982874 | C/T | *TBL2/MLXIPL* | 3downstream | T:0.04673 | T:0.113 | T:0.113 | T:0.098 |
| rs174546 | 11 | 61569830 | C/T | *FADS2* | intronic | C:0.1866 | T:0.367 | T:0.394 | T:0.034 |
| rs1832007 | 10 | 5254847 | A/G | *AKR1C4* | intronic | G:0.222 | G:0.125 | G:0.113 | G:0.009 |
| rs1936800 | 6 | 127436064 | C/T |  | upstream gene | C:0.5444 | T:0.491 | T:0.495 | T:0.557 |
| rs2036402 | 5 | 156464242 | T/C | *HAVCR1* | intronic | C:0.4579 | C:0.292 | C:0.162 | C:0.026 |
| rs2068888 | 10 | 94839642 | G/A | *CYP26A1* | 3downstream | A:0.4272 | A:0.472 | A:0.673 | A:0.239 |
| rs2131925 | 1 | 63025942 | G/T | *DOCK7* | intronic | T:0.5462 | T:0.668 | T:0.804 | T:0.302 |
| rs2228603 | 19 | 19329924 | C/T | *NCAN* | coding nonsyn | T:0.02347 | T:0.062 | T:0.067 | T:0 |
| rs2286276 | 7 | 72987354 | C/T | *TBL2/MLXIPL* | intronic | T:0.09906 | T:0.230 | T:0.124 | T:0.397 |
| rs2412710 | 15 | 42683787 | G/A | *CAPN3* | intronic | A:0.01415 | A:0.025 | A:0.003 | A:0.049 |
| rs2929282 | 15 | 44245931 | A/T | *FRMD5* | intronic | T:0.1355 | T:0.048 | T:0.041 | T:0.339 |
| rs2954029 | 8 | 126490972 | A/T | *RP11-136O12.2* | non-coding intronic | T:0.3732 | T:0.433 | T:0.539 | T:0.273 |
| rs3198697 | 16 | 15129940 | C/T | *PDXDC1* | coding syn | T:0.1244 | T:0.387 | T:0.018 | T:0.037 |
| rs3764261 | 16 | 56993324 | C/A | *AC012181.1* | 3downstream | T:0.4019 | A:0.281 | A:0.193 | A:0.345 |
| rs442177 | 4 | 88030261 | G/T | *AFF1* | intronic | T:0.7038 | T:0.567 | T:0.570 | T:0.466 |
| rs4722551 | 7 | 25991826 | T/C |  | upstream gene | C:0.1846 | C:0.193 | C:0.041 | C:0.023 |
| rs4765127 | 12 | 124460167 | G/T | *ZNF664* | non-coding intronic | 0.229 | T:0.383 | T:0.121 | T:0.351 |
| rs4846914 | 1 | 230295691 | G/A | *GALNT2* | non-coding intronic | A:0.3538 | A:0.569 | A:0.222 | A:0.083 |
| rs6065906 | 20 | 44554015 | T/C |  | regulatory region | C:0.03738 | C:0.194 | C:0.039 | C:0.164 |
| rs645040 | 3 | 135926622 | G/T | *RP11-463H24.1* | 5upstream | G:0.1533 | n.a. | n.a. | n.a. |
| rs6831256 | 4 | 3473139 | A/G | *DOK7* | non-coding intronic | G:0.5924 | G:0.406 | G:0.371 | G:0.609 |
| rs6882076 | 5 | 156390297 | T/C | *TIMD4* | 5upstream | C:0.8685 | C:0.677 | C:0.781 | C:0.330 |
| rs7248104 | 19 | 7224431 | G/A | *INSR* | intronic | A:0.3122 | A:0.419 | A:0.366 | A:0.316 |
| rs731839 | 19 | 33899065 | G/A | *PEPD* | intronic | A:0.5771 | A:0.675 | A:0.497 | A:0.632 |
| rs8077889 | 17 | 41878166 | A/C | *MPP3* | 3downstream | C:0.08451 | C:0.239 | C:0.059 | C:0.149 |
| rs964184 | 11 | 116648917 | G/C | *APOA5/BUD13* | 3utr | C:0.722 | C:0.839 | C:0.740 | C:0.813 |
| rs9686661 | 5 | 55861786 | C/T | *AC022431.2* | intronic | T:0.1869 | T:0.178 | T:0.101 | T:0.241 |
| rs998584 | 6 | 43757896 | C/A |  | downstream | T:0.6495 | A:0.530 | A:0.577 | A:0.173 |
| SNPs are ordered by rs ID. Base pairs correspond to build 37. Alleles are in forward strand. Allelic frequencies were calculated based on the results of this study (normotriglyceridemic group) or the 1000 Genomes Project. **n.a.** Not available. This set of variants has been previously associated with TG levels, as reported by Willer *et al*. 2013 (32) and Weissglass-Volkov *et al*. 2013 (33) . | | | | | | | | | |

| **Supplementary Table 2. Association of the total set of TG-related known genetic variants with the risk of FHTG and CHTG.** | | | | | | | |
| --- | --- | --- | --- | --- | --- | --- | --- |
|  |  |  |  | **FTHG** | | **CHTG** | |
| **CHR** | **SNP** | **BP** | **EA** | **OR [95% CI]** | ***P*** | **OR [95% CI]** | ***P*** |
| 11 | rs964184 | 116648917 | G | **9.6 [4.1-22.8]** | **2.54x10^-07^** | **1.862[1.559-2.225]** | **7.66x10^-12^** |
| 15 | rs1532085 | 58683366 | A | **2.54 [1.32-4.91]** | **0.0055** | 1.057[0.89-1.255] | 0.5285 |
| 19 | rs7248104 | 7224431 | A | **0.4 [0.21-0.78]** | **0.0071** | 0.9338[0.7842-1.112] | 0.4416 |
| 2 | rs1260326 | 27730940 | T | **2.27 [1.17-4.39]** | **0.0155** | **1.287[1.081-1.533]** | **0.004642** |
| 4 | rs6831256 | 3473139 | A | **1.97 [1.09-3.57]** | **0.0247** | 0.9506[0.8024-1.126] | 0.558 |
| 20 | rs6065906 | 44554015 | C | **3.6 [1.13-11.5]** | **0.0304** | 1.33[0.9633-1.836] | 0.08311 |
| 10 | rs2068888 | 94839642 | A | **0.52 [0.29-0.96]** | **0.0366** | 1.046[0.8863-1.235] | 0.5936 |
| 7 | rs2286276 | 72987354 | A | **0.29 [0.09-0.93]** | **0.0375** | 0.808[0.6303-1.036] | 0.09242 |
| 8 | rs2954029 | 126490972 | T | 0.57 [0.31-1.05] | 0.0704 | 0.8798[0.7368-1.051] | 0.157 |
| 6 | rs998584 | 43757896 | C | 0.54 [0.26-1.12] | 0.0982 | 0.8952[0.7561-1.06] | 0.199 |
| 8 | rs12678919 | 19844222 | G | 0.04 [0.0009-2.17] | 0.1162 | **0.6635[0.4344-1.013]** | **0.0577** |
| 19 | rs10401969 | 19407718 | C | 0.27 [0.05-1.44] | 0.1239 | **0.6054[0.3909-0.9376]** | **0.02455** |
| 6 | rs1936800 | 127436064 | C | 0.63 [0.34-1.17] | 0.1413 | 1.043[0.8788-1.239] | 0.6277 |
| 16 | rs3764261 | 56993324 | T | 0.64 [0.34-1.2] | 0.1658 | 0.8724[0.7334-1.038] | 0.1234 |
| 1 | rs4846914 | 230295691 | G | 1.46 [0.83-2.58] | 0.1879 | 1.086[0.9151-1.289] | 0.3444 |
| 17 | rs8077889 | 41878166 | C | 0.39 [0.09-1.66] | 0.2038 | 1.09[0.8203-1.448] | 0.5523 |
| 12 | rs4765127 | 124460167 | T | 1.56 [0.78-3.13] | 0.2104 | 0.9385[0.7698-1.144] | 0.5302 |
| 10 | rs1832007 | 5254847 | G | 0.63 [0.3-1.34] | 0.2293 | 0.9802[0.7994-1.202] | 0.8476 |
| 5 | rs9686661 | 55861786 | T | 0.62 [0.29-1.35] | 0.2319 | 1.092[0.8837-1.35] | 0.4144 |
| 5 | rs2036402 | 156464242 | G | 0.69 [0.37-1.28] | 0.2396 | 1.079[0.9067-1.283] | 0.3931 |
| 19 | rs2228603 | 19329924 | T | 0.12 [0.003-4.29] | 0.2439 | 0.6387[0.368-1.109] | 0.111 |
| 7 | rs4722551 | 25991826 | C | 0.64 [0.27-1.51] | 0.3071 | 1.006[0.8088-1.252] | 0.9543 |
| 3 | rs645040 | 135926622 | G | 0.65 [0.28-1.49] | 0.3078 | 0.8727[0.7078-1.076] | 0.2022 |
| 15 | rs2412710 | 42683787 | A | 2.47 [0.42-14.64] | 0.3197 | 0.9771[0.5328-1.792] | 0.9402 |
| 1 | rs12748152 | 27138393 | T | 0.35 [0.04-2.79] | 0.3237 | 1.294[0.7953-2.106] | 0.2993 |
| 7 | rs17145738 | 72982874 | T | 0.29 [0.03-3.36] | 0.3247 | 0.945[0.6545-1.364] | 0.7625 |
| 5 | rs6882076 | 156390297 | T | 0.67 [0.29-1.55] | 0.3529 | 0.9681[0.7822-1.198] | 0.7653 |
| 16 | rs11649653 | 30918487 | G | 0.76 [0.41-1.39] | 0.3667 | **0.8281[0.6883-0.9964]** | **0.0457** |
| 19 | rs731839 | 33899065 | G | 1.21 [0.64-2.27] | 0.5545 | 1.002[0.8429-1.191] | 0.9832 |
| 16 | rs3198697 | 15129940 | T | 1.3 [0.54-3.09] | 0.559 | 0.8461[0.6686-1.071] | 0.164 |
| 16 | rs1121980 | 53809247 | T | 1.24 [0.59-2.62] | 0.5674 | NA | NA |
| 12 | rs11613352 | 57792580 | T | 0.87 [0.49-1.56] | 0.6431 | **0.7934[0.6708-0.9384]** | **0.006869** |
| 4 | rs442177 | 88030261 | C | 0.88 [0.45-1.74] | 0.7195 | 0.9389[0.7826-1.126] | 0.4971 |
| 15 | rs2929282 | 44245931 | T | 0.87 [0.31-2.42] | 0.7834 | 0.9658[0.7345-1.27] | 0.8034 |
| 1 | rs2131925 | 63025942 | G | 1.08 [0.58-2] | 0.8097 | 0.9567[0.8076-1.133] | 0.6084 |
| 1 | rs10889337 | 62980607 | A | 1.05 [0.56-1.97] | 0.888 | 0.9241[0.7791-1.096] | 0.3643 |
| 11 | rs174546 | 61569830 | C | 1.04 [0.46-2.39] | 0.9211 | NA | NA |
| 10 | rs10761731 | 65027610 | T | 1.03 [0.54-1.96] | 0.9278 | **0.7698[0.6201-0.9556]** | **0.01771** |
| SNPs are ordered by *p* value in FHTG. Logistic regression corrected for sex, age, age^2^, BMI, T2D and ancestry. **EA**: Effect allele. | | | | | | | |

| **Supplementary Table 3. Monogenic component set of mutations analyzed in this study.** | | | | | | | | | | | | | |
| --- | --- | --- | --- | --- | --- | --- | --- | --- | --- | --- | --- | --- | --- |
| **GENE** | **CHR** | **POS** | **RS ID** | **REF/**  **ALT** | **HGVS c.** | **HGVS p.** | **Sequence ontology** | ***f* This study** | ***f* AFR** | ***f* EAS** | ***f* EUR** | ***f* SAS** |  |
| LPL | 8 | 19805815 | rs11542065 | C/G | c.213C>G | p.His71Gln | Missense | 0 | 0.012549 | 0 | 0 | 0.000065 |  |
| LPL | 8 | 19811679 | rs372668179 | G/A | c.590G>A | p.Arg197His | Missense | 0.001497 | 0.00004005 | 0.0001 | 0 | 0 |  |
| LPL | 8 | 19811710 | rs118204076 | C/G | c.621C>G | p.Asp207Glu | Missense | 0 | 0.000062 | 0.00005012 | 0 | 0 |  |
| LPL | 8 | 19811733 | rs118204057 | G/A | c.644G>A | p.Gly215Glu | Missense | 0 | 0.000062 | 0 | 0.000334 | 0.000065 |  |
| LPL | 8 | 19811751 | rs118204061 | T/C | c.662T>C | p.Ile221Thr | Missense | 0 | 0 | 0.000054 | 0.000018 | 0 |  |
| LPL | 8 | 19811769 | rs528243561 | T/C | c.680T>C | p.Val227Ala | Missense | 0 | 0 | 0 | 0 | 0 |  |
| LPL | 8 | 19819628 | rs116403115 | T/G | c.1325T>G | p.Val442Gly | Missense | 0 | 0.000062 | 0 | 0.000273 | 0.000196 |  |
| GPIHBP1 | 8 | 144294674 | rs72691625 | G/A | g.-469G>A | - | 5upstream | 0.266 | 0.05159 | 0.1864 | 0.191 | 0 |  |
| GPIHBP1 | 8 | 144296900 | rs587777638 | G/A | c.194G>A | p.Cys65Tyr | Missense | 0 | 0 | 0 | 0.000012 | 0.000037 |  |
| GPIHBP1 | 8 | 144296908 | rs587777639 | T/G | c.202T>G | p.Cys68Gly | Missense | 0 | 0 | 0 | 0.000022 | 0 |  |
| GPIHBP1 | 8 | 144297158 | rs587777643 | C/G | c.320C>G | p.Ser107Cys | Missense | 0 | 0 | 0 | 0.000009 | 0.000033 |  |
| GPIHBP1 | 8 | 144297161 | rs752728823 | C/T | c.323C>G | p.Thr108Arg | Missense | 0 | 0 | 0 | 0.000018 | 0.000392 |  |
| GPIHBP1 | 8 | 144297182 | rs587777637 | A/C | c.344A>C | p.Gln115Pro | Missense | 0 | 0 | 0 | 0 | 0 |  |
| GPIHBP1 | 8 | 144297206 | rs201685731 | G/A | c.368G>A | p.Gly123Glu | Missense | 0 | 0 | 0 | 0.000421 | 0.000065 |  |
| GPIHBP1 | 8 | 144297269 | rs78367243 | C/T | c.431C>T | p.Ser144Phe | Missense | 0 | 0.161889 | 0 | 0.000519 | 0.000098 |  |
| GPIHBP1 | 8 | 144297361 | rs145844329 | G/C | c.523G>C | p.Gly175Arg | Missense | 0 | 0.005245 | 0 | 0.000525 | 0 |  |
| APOA5 | 11 | 116661001 | rs143292359 | G/A | c.944C>T | p.Ala315Val | Missense | 0 | 0.000062 | 0 | 0.000713 | 0.000065 |  |
| APOA5 | 11 | 116661656 | rs201079485 | G/A | c.289C>T | p.Gln97Ter | Nonsense | 0 | 0.000193 | 0 | 0.000107 | 0 |  |
| LMF1 | 16 | 904551 | rs4984948 | G/C | c.1685C>G | p.Pro562Arg | Missense | 0.178 | 0.006047 | 0.137285 | 0.006827 | 0.050174 |  |
| LMF1 | 16 | 904698 | rs748287562 | C/T | c.1538G>A | p.Arg513Gln | Missense | 0 | 0.000159 | 0.000062 | 0.000052 | 0 |  |
| LMF1 | 16 | 919894 | rs181731943 | C/T | c.1405G>A | p.Ala469Thr | Missense | 0 | 0.000067 | 0.000168 | 0.000461 | 0.000394 |  |
| LMF1 | 16 | 919948 | rs138205062 | G/A | c.1351C>T | p.Arg451Trp | Missense | 0 | 0.000654 | 0.000167 | 0.00324 | 0.008175 |  |
| LMF1 | 16 | 919982 | rs121909397 | G/C | c.1317C>G | p.Tyr439Ter | Nonsense | 0 | 0 | 0.00039 | 0.000107 | 0.000098 |  |
| LMF1 | 16 | 920007 | rs115416993 | C/A | c.1292C>A | p.Ala431Asp | Missense | 0 | 0.025407 | 0 | 0.000045 | 0.000033 |  |
| LMF1 | 16 | 920733 | rs199713950 | C/T | c.1228G>A | p.Gly410Arg | Missense | 0 | 0 | 0.006124 | 0.00016 | 0.000065 |  |
| LMF1 | 16 | 920870 | rs35168378 | G/A | c.1091G>A | p.Arg364Gln | Missense | 0.016 | 0.035494 | 0.000396 | 0.029321 | 0.026423 |  |
| LMF1 | 16 | 921173 | rs199615983 | C/T | c.1066G>A | p.Glu356Lys | Missense | 0 | 0.000075 | 0.000059 | 0.000247 | 0 |  |
| LMF1 | 16 | 921179 | rs143076454 | C/T | c.1060C>T | p.Arg354Trp | Missense | 0.014 | 0.003892 | 0 | 0.018472 | 0.007568 |  |
| LMF1 | 16 | 929572 | rs554054538 | G/A | c.895C>T | p.Gln299Ter | Nonsense | 0 | 0 | 0 | 0.000072 | 0 |  |
| LMF1 | 16 | 929677 | rs777579889 | G/C | c.790C>T | p.Arg264Cys | Missense | 0 | 0 | 0 | 0 | 0 |  |
| LMF1 | 16 | 929680 | rs746165846 | G/A | c.787C>T | p.His263Tyr | Missense | 0.001497 | 0 | 0 | 0 | 0 |  |
| LMF1 | 16 | 943047 | rs192224688 | C/T | c.689G>A | p.Arg230Gln | Missense | 0 | 0.00614 | 0.000178 | 0.00038 | 0.000087 |  |
| LMF1 | 16 | 943053 | rs754772870 | G/A | c.683G>A | p.Gly228Glu | Missense | 0 | 0 | 0 | 0.000531 | 0 |  |
| APOC2 | 19 | 45451743 | rs148343756 | C/T | c.8C>T | p.Thr3Ile | Missense | 0 | 0.003814 | 0 | 0.000062 | 0 |  |
| APOC2 | 19 | 45451745 | rs202190413 | C/G | c.10C>G | p.Arg4Gly | Missense | 0 | 0 | 0 | 0.000105 | 0.000033 |  |
| APOC2 | 19 | 45452024 | rs120074114 | A/C | c.122A>C | p.Lys41Thr | Missense | 0 | 0.000062 | 0 | 0.001292 | 0.000131 |  |
| SNPs are ordered by gene and rs ID. Base pairs correspond to build 37. Alleles are in forward strand. Allelic frequencies were calculated based on the results of this study (normotriglyceridemic group) or the GnomAD Project. **n.a.** Not available. This set of variants was previously associated with FCS and FHTG as reported by Serveaux Dancer 2018 and Dron 2019. | | | | | | | | | | | | | |

**Supplementary Table 4. Prevalence of known FCS monogenic variants.**

| **GENE** | | **CHR** | **POS** | **SNP** | **REF/ALT** | **HGVS p.** | **Sequence ontology** | **MAF**  **NTG** | **MAF**  **CHTG** | **MAF**  **FHTG** | **Reference** |
| --- | --- | --- | --- | --- | --- | --- | --- | --- | --- | --- | --- |
| *LPL* | | 8 | 19805815 | rs11542065 | C/G | p.His71Gln | Missense | 0 | 0 | 0 | Dron, 2019. |
|  | |  | 19811679 | rs372668179 | G/A | p.Arg197His | Missense | 0.001497 | n.a. | 0 | Dron, 2019. |
|  | |  | 19811710 | rs118204076 | C/G | p.Asp207Glu | Missense | 0 | n.a. | 0 | Dron, 2019. |
|  | |  | 19811733 | rs118204057 | G/A | p.Gly215Glu | Missense | 0 | 0 | 0 | Dron, 2019. |
|  | |  | 19811751 | rs118204061 | T/C | p.Ile221Thr | Missense | 0 | 0 | 0 | Dron, 2019. |
|  | |  | 19811769 | rs528243561 | T/C | p.Val227Ala | Missense | 0 | n.a. | 0.005747 | Dron, 2019. |
|  | |  | 19819628 | rs116403115 | T/G | p.Val442Gly | Missense | 0 | n.a. | 0 | Dron, 2019. |
| *GPIHBP1* | | 8 | 144294674 | rs72691625 | G/A | - | - | 0.266 | 0.243 | 0.262 |  |
|  | |  | 144296900 | rs587777638 | G/A | p.Cys65Tyr | Missense | 0 | n.a. | 0 |  |
|  | |  | 144296908 | rs587777639 | T/G | p.Cys68Gly | Missense | 0 | n.a. | 0 |  |
|  | |  | 144297158 | rs587777643 | C/G | p.Ser107Cys | Missense | 0 | n.a. | 0 |  |
|  | |  | 144297161 | rs752728823 | C/T | p.Thr108Met | MIssense | 0 | n.a. | 0 |  |
|  | |  | 144297182 | rs587777637 | A/C | p.Gln115Pro | Missense | 0 | n.a. | 0 |  |
|  | |  | 144297206 | rs201685731 | G/A | p.Gly123Glu | Missense | 0 | n.a. | 0 | Dron, 2019. |
|  | |  | 144297269 | rs78367243 | C/T | p.Ser144Phe | Missense | 0 | 0.004831 | 0 |  |
|  | |  | 144297361 | rs145844329 | G/C | p.Gly175Arg | Missense | 0 | 0.001174 | 0 | Dron, 2019. |
| *APOA5* | | 11 | 116661001 | rs143292359 | G/A | p.Ala315Val | Missense | 0 | 0. | 0 | Dron, 2019. |
|  | |  | 116661656 | rs201079485 | G/A | p.Gln97Ter | Nonsense | 0 | n.a. | 0 | Dron, 2019. |
| *LMF1* | | 16 | 904551 | rs4984948 | C/G | p.Pro562Arg | Missense | 0.178 | 0.2343 | 0.259 | Seveaux Dancer, 2018. |
|  | |  | 904698 | rs748287562 | C/T | p.Arg513Gln | Missense | 0 | n.a. | 0 |  |
|  | |  | 919894 | rs181731943 | G/A | p.Ala469Thr | Missense | 0 | 0.002415 | 0 | Dron, 2019. |
|  | |  | 919948 | rs138205062 | G/A | p.Arg451Trp | Missense | 0 | 0 | 0 | Dron, 2019. |
|  | |  | 919982 | rs121909397 | G/C | p.Tyr439Ter | Nonsense | 0 | 0.001174 | 0 | Dron, 2019. |
|  | |  | 920007 | rs115416993 | C/A | p.Ala431Asp | Missense | 0 | 0.001208 | 0 | Seveaux Dancer, 2018. |
|  | |  | 920733 | rs199713950 | C/T | p.Gly410Arg | Missense | 0 | n.a. | 0 | Dron, 2019. |
|  | |  | 920870 | rs35168378 | G/A | p.Arg364Gln | Missense | 0.016 | 0.007042 | 0.048 | Seveaux Dancer, 2018. |
|  | |  | 921173 | rs199615983 | C/T | p.Glu356Lys | Missense | 0 | n.a. | 0 |  |
|  | |  | 921179 | rs143076454 | C/T | p.Arg354Trp | MIssense | 0.014 | 0.005869 | 0.024 | Seveaux Dancer, 2018. |
|  | |  | 929572 | rs554054538 | G/A | p.Gln299Ter | Nonsense | 0 | n.a. | 0 |  |
|  | |  | 929677 | rs777579889 | G/A | p.Arg264Cys | Missense | 0 | n.a. | 0 |  |
|  | |  | 929680 | rs746165846 | G/A | p.His263Tyr | Missense | 0.001497 | n.a. | 0 | Dron, 2019. |
|  | |  | 943047 | rs192224688 | C/T | p.Arg230Gln | Missense | 0 | 0 | 0 |  |
|  | |  | 943053 | rs754772870 | G/A | p.Gly228Glu | Missense | 0 | n.a. | 0 | Dron, 2019. |
| *APOC2* | | 19 | 45451743 | rs148343756 | C/T | p.Thr3Ile | Missense | 0 | 0 | 0 | Dron, 2019. |
|  | |  | 45451745 | rs202190413 | C/G | p.Arg4Gly | Missense | 0 | n.a. | 0 | Dron, 2019. |
|  | |  | 45452024 | rs120074114 | A/C | p.Lys41Thr | Missense | 0 | 0 | 0 | Dron, 2019. |
|  | SNPs are ordered by chr and pos. Base position correspond to build 37. **NL:** Normotriglyceridemic. **FHTG**: Familiar hypertriglyceridemia. | | | | | | | | | | |

| 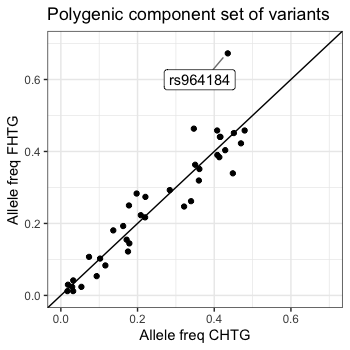 | 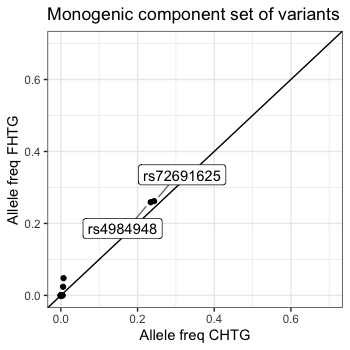 |
| --- | --- |
| **Supplementary Figure 1. Comparison of allele frequencies between CHTG and FHTG individuals.** | |
