# Supplementary material for: Familial hypertriglyceridemia: an entity with distinguishable features from other causes of hypertriglyceridemia
Source: Lipids Health Dis. 2021 Feb 15;20:14. doi: 10.1186/s12944-021-01436-6 (PMC7885394; doi:10.1186/s12944-021-01436-6)
Supplement: Supplementary file 2 — Additional file 2. [file 12944_2021_1436_MOESM2_ESM.docx]

**Supplementary Table 5. Clinical and biochemical characteristics of study subjects by T2D status.**

|  | **Normal Triglycerides** | | **CHTG** | | **FHTG** | |
| --- | --- | --- | --- | --- | --- | --- |
|  | **Non T2D** | **T2D** | **Non T2D** | **T2D** | **Non T2D** | **T2D** |
| Sample size | 415 | 194 | 438 | 290 | 44 | 40 |
| Sex (% females) ^a^ | 70.6 | 67.5 | 47 | 57 | 26.6 | 18.2 |
| Age (years) | 53^a^  [48-61] | 58  [49 -66] | 51^a^  [48-59] | 56  [47-62] | 42.7  [35-47] | 43.5  [38-50 ] |
| BMI (kg/m^2^) | 27  [24.7-30] | 27.3  [24.3-30.3] | 28.4  [25.6-31.1] | 28.8  [26.1-31.9] | 28.9  [26.1-32.1] | 28.8  [26-32.2] |
| Waist circumference (cm) | 90^a^  [84-97.7] | 94  [85.5-100] | 95^a^  [89-101] | 98.7  [90-107] | 96.2  [87.5-100.5] | 96  [89-102] |
| Triglycerides (mg/dl) | 112  [84-130] | 113  [ 89-131] | 269  [228-331] | 276  [228-333] | 930  [267-1466.93] | 712  [355-1255] |
| Cholesterol (mg/dl) | 176^a^  [152-199] | 202  [ 179-223] | 210^a^  [182-232] | 221  [194-248] | 199  [162-251] | 186  [168-257] |
| HDL-C (mg/dl) | 48^a^  [42-57] | 45  [39-51] | 39^a^  [33-45] | 35  [30-41] | 33  [28-39] | 29.6  [22.9-34.9] |
| Non- HDL-C ( mg/dl) | 126^a^  [107-153] | 153  [127-154] | 172^a^  [148-193] | 181  [159-203] | 173  [127-216] | 148  [ 132-231] |
| Apo B (mg/dl) | 102  [86-118] | 105  [90-119] | 126  [108-142] | 128  [106-148] | 91  [82-97] | 93.3  [86.8-99.7] |
| Glucose (mg/dl) | 90^a^  [83-108] | 135  [113-166] | 87^a^  [81-93] | 180  [130-245] | 94^a^  [86-102] | 139  [126-185] |
| Insulin (μU/l) | 7.8^a^  [5.4-11.5] | 13.4  [10.4-17.5] | 9.2  [6.9-13.7] | 10.5  [7.8-15.9] | 13  [7.9-18.8] | 13  [8.2-20.2] |
| HOMA2 B% | 108 ^a^  [85-139] | 66  [44-135] | 129  [104-168] | 35^a^  [14-70] | 113 ^a^  [69-161] | 53  [26-104] |
| HOMA IR | 1.6^a^  [1.1-2.4] | 3.6  [2.7-8.9] | 1.2  [1.5-3.5] | 4.1^a^  [3.1-6.1] | 3^a^  [1.8-4.4] | 4.4  [3.8-7.7] |
| AP (%)* | 0 | 0 | 0 | 0 | 9.5^a^ | 42.8 |
| Median [25^th^ percentile-75^th^ percentile] or percentages are shown. AP: Acute pancreatitis by hypertriglyceridemia  Chi square/Fisher exact test; U Mann Whitney Test for metabolic traits adjusted for age and sex ; ^a^ p< 0.001 | | | | | | |
